# Neural substrates of ambiguity and beauty in haiku poetry: an fMRI study

**DOI:** 10.3389/fnhum.2026.1887834

**Published:** 2026-08-28

**Authors:** Jimpei Hitsuwari, Michio Nomura

**Affiliations:** 1 Experimental Psychology Unit, Faculty of Humanities and Social Sciences, Helmut Schmidt University/University of the Federal Armed Forces Hamburg, Hamburg, Germany; 2 Japan Society for the Promotion of Science, Tokyo, Japan; 3 Graduate School of Education, Kyoto University, Kyoto, Japan

**Keywords:** aesthetic experience, ambiguity, fMRI, haiku poetry, neuroaesthetics

## Abstract

Although neuroaesthetics has extensively examined visual art and music, the neural mechanisms underlying poetic appreciation remain less explored. This fMRI study investigated how the brain processes ambiguity and aesthetic experiences using haiku, the world’s shortest form of poetry. Originating in Japan, haiku is characterized by extreme brevity, often omitting information to compel readers to generate mental imagery and confront inherent ambiguities. Thirty-nine participants read haiku, which were pre-categorized via an independent survey into high- and low-ambiguity conditions, and rated their beauty inside the scanner. Consistent with previous findings, the behavioral results indicated a preference for low-ambiguity haiku (a small effect, standardized *β* = −0.16). Neuroimaging revealed that reading low-ambiguity haiku, compared to high-ambiguity ones, elicited greater activation in the posterior cingulate cortex and the left inferior parietal lobule, including the angular gyrus (cluster-level pFWE = 0.033 and 0.005, respectively). These regions are associated with the default mode network and semantic integration, suggesting that more interpretable poems may involve stronger self-referential processing and scene construction. Higher beauty ratings were significantly associated with increased activation in the primary visual cortex (cluster-level pFWE = 0.002). Our findings imply that the aesthetic appeal of haiku depends on semantic accessibility, with reduced ambiguity associated with greater engagement of self-referential processing in the default mode network and with increased activity in the visual cortex. More broadly, using haiku as a model offers a foundation for neuroscientific investigations of ambiguity and aesthetic experience across poetry, narratives, and other art forms.

## 1 Introduction

### 1.1 Neuroaesthetics and poetry

Since the early 2000s, the field of neuroaesthetics, which empirically examines aesthetic and artistic experiences using neuroimaging methods, has developed rapidly (Chatterjee and Vartanian, 2014; Jacobsen et al., 2006; Kawabata and Zeki, 2004). The research spans diverse domains, including paintings (Durkin et al., 2025), other image stimuli (Arioli et al., 2025; Jacobsen et al., 2006), music (Brattico et al., 2025; Koelsch, 2014), and dance (Calvo-Merino et al., 2005; Cross, 2025). Cross-domain studies have also been conducted; for example, Ishizu and Zeki (2011) demonstrated that the medial orbitofrontal cortex (mOFC) is activated when experiencing beauty in paintings and music. The involvement of the default mode network (DMN) has also been repeatedly shown across artistic domains, particularly when aesthetic appreciation involves interpretive ambiguity, sense-making, and the integration of complex or semantically rich information (Vessel and Ovadia, 2025). However, Hu et al. (2020) failed to identify brain regions that were consistently associated with aesthetic judgments across faces and visual art, suggesting that a single “beauty center” may not exist. Although the humanities have long considered poetry a distinctive form of thought and knowledge (e.g., Langione, 2016), neuroscientific research on linguistic arts such as poetry remains limited (Chaudhuri and Bhattacharya, 2025a). Nonetheless, studies in this area have increased in recent years.

In a pioneering study (Zeman et al., 2013), participants appreciated poetry and prose inside an MRI scanner, revealing that poetry appreciation elicited greater activation in the right posterior-to-middle cingulate cortex, left superior temporal gyrus, and bilateral hippocampus. These regions are part of or related to the DMN, suggesting that reading poetry may strongly evoke internal processes such as autobiographical memory retrieval, introspective thought, and mentalizing (Zeman et al., 2013). Gao and Guo (2018) found that appreciating classical Chinese poetry broadly recruited the visual and linguistic semantic processing necessary for reading poetry, including visual processing (occipital lobe), word recognition and visual word form processing (fusiform gyrus), sentence comprehension and semantic processing (inferior frontal gyrus and temporal regions), and rhythm and prosody processing (middle frontal lobe). Studies using German poetry and proverbs (Bohrn et al., 2013; Wassiliwizky et al., 2017) have demonstrated the activation of reward-related brain regions such as the caudate nucleus and nucleus accumbens. A recent study (Kumar et al., 2025) found that comprehending meaningful Sanskrit verses engaged a broad network involving the occipital pole, inferior temporal gyrus, middle temporal gyrus, and orbitofrontal cortex, indicating that poetic language comprehension is multimodal, involving visual imagery, semantic integration, and higher-order evaluation in addition to auditory processing.

Poetry appreciation has also been investigated using neuroscience methods other than functional magnetic resonance imaging (fMRI). Obermeier et al. (2016) revealed that stanzas with meters and rhymes showed smaller event-related potential (ERP) responses than stanzas without meters or rhymes, specifically the N400, which responds to semantic incongruity, and the P600, which is related to grammatical processing and syntactic parsing. Chen et al. (2016) demonstrated that rhyme violations in classical Chinese poetry increased the N400-like negative component centered on the right temporal region, whereas semantic violations increased the typical N400 components from the central to posterior regions.

Neuroscientific research on poetry appreciation has revealed the involvement of multifaceted processes, including visual processing, introspective thought, reward processing, and prosodic-semantic processing. However, such fMRI and electroencephalography (EEG) studies remain limited. In this context, the present study conducted an fMRI experiment using haiku, a short poetic form with a strict structure well suited for psychological experiments (Chaudhuri and Bhattacharya, 2025a; Nasemann et al., 2023).

### 1.2 Aesthetic appreciation of haiku poetry

Haiku is the world’s shortest poetic form, originating in Japan (Shirane, 2023). It is enjoyed in other languages, including English, and has been widely examined internationally as a research subject (Belfi et al., 2018; Blasko and Merski, 1998; Mehl et al., 2025). Eye-movement studies, for example, have examined how readers process English-language haiku, demonstrating that its structural characteristics shape reading behavior (Geyer et al., 2020; Müller et al., 2017). Its defining characteristic lies in the fixed structure composed of 5-7-5 syllables, with poem length and rhythm nearly controlled, making it ideal for psychological experiments (Chaudhuri and Bhattacharya, 2025b; Niikuni et al., 2022). The aesthetic evaluation of haiku has been shown to be predicted by imagery vividness (Belfi et al., 2018; Blasko and Merski, 1998; Hitsuwari and Nomura, 2022; Mehl et al., 2025), basic positive emotions, higher-order emotions such as awe and nostalgia (Hitsuwari and Nomura, 2024a), and familiarity (Blasko and Merski, 1998). Simultaneously, individual traits such as imagery ability [visual (Belfi et al., 2018; Hitsuwari and Nomura, 2022), auditory (Chaudhuri and Bhattacharya, 2025b), and olfactory (Hitsuwari et al., 2023a)], openness to experience (Chaudhuri and Bhattacharya, 2025b; Hitsuwari and Nomura, 2022), and attitudes toward ambiguity (Hitsuwari and Nomura, 2025a) have also been shown to have direct and indirect (through interactions with other variables) influences on haiku evaluation. More recently, with advances in generative AI, poem authorship—that is, whether a poem is written by a human or generated by AI—has emerged as another factor influencing aesthetic evaluation. Recent studies have compared readers’ aesthetic judgments of human- and AI-authored haiku (Hitsuwari et al., 2023b) and other literary texts (Guan and Rong, 2026). The Neurocognitive Poetics Model (Jacobs, 2015) provides a broader theoretical framework for the aesthetic appreciation of literary texts, proposing that foregrounded features such as interpretive ambiguity slow processing and engage readers in the active construction of meaning gestalts, thereby giving rise to aesthetic feelings. The present study situates the interpretive ambiguity of haiku within this framework.

Among these factors, the present study focuses on the ambiguity of haiku. Because haiku is the world’s shortest poetic form, the amount of information presented is limited, leaving considerable room for reader interpretation. While haiku poets and critics advocate for the role of ambiguity in haiku (Gilbert, 2023; Kishimoto, 2008), empirical research has revealed a more complex relationship between ambiguity and aesthetic evaluation. For example, Hitsuwari and Nomura (2024a) presented Japanese haiku to Japanese participants and German haiku to German participants, to evaluate ambiguity and beauty; the results showed that in both samples, ambiguity decreased the beauty ratings of haiku. This relationship showed an interaction with culture, with the degree to which ambiguity decreased beauty being more moderate among the Japanese participants. In addition, Hitsuwari and Nomura (2024b) found that experts were less likely to perceive haiku as ambiguous compared to novices and did not lower their evaluation of the haiku. These studies demonstrate that the relationship between ambiguity and aesthetic evaluation depends on the culture and expertise level of the evaluator, but collectively indicate that aesthetic evaluation tends to increase when ambiguity is lower. Conversely, this suggests that the beauty of haiku is more likely to increase when fluency is high (Obermeier et al., 2016) or when clear images can be represented (Belfi et al., 2018). Consistent with this, Maruyama and Ishizu (2025) reported that descriptive titles, which make Japanese poems easier to understand, elicited greater reflection than interpretive titles, suggesting that increased processing fluency may enhance aesthetic engagement with poetry. Beyond imagery, processing fluency, and individual differences, formal and phonological features of Japanese poetry also influence aesthetic evaluation. Yoshio and Kambara (2025) reported that metric structure increased the perceived beauty of Japanese poems, indicating that the aesthetic appreciation of short Japanese verse is shaped not only by semantic content but also by its sound-based form.

To our knowledge, no fMRI studies of haiku exist, although several EEG studies have been conducted (Chaudhuri and Bhattacharya, 2025a; Meshcherina et al., 2026; Nasemann et al., 2023). For example, Chaudhuri and Bhattacharya (2025a) showed that aesthetic appeal increases when the left visual processing areas are activated, suggesting the importance of visual image generation and spatial processing in haiku appreciation, and supporting behavioral data showing a positive relationship between mental imagery and aesthetic evaluation. More recently, Meshcherina et al. (2026) used EEG and machine learning to predict aesthetic evaluations of structurally matched haiku and senryu from oscillatory neural activity, showing that aesthetic evaluation is content-specific and shaped by anticipatory neural states. These EEG studies characterize the temporal and spectral dynamics of poetic appreciation but leave open where in the brain interpretive ambiguity and beauty are represented, which motivates the present fMRI approach.

### 1.3 Ambiguity in the brain

Empson’s classical definition of ambiguity is widely used in the field of literary criticism. Empson (1949) conceived of ambiguity as a concept extending beyond everyday polysemy, defining it as “any verbal nuance, however slight, which gives room for alternative reactions to the same piece of language (p. 1).” Zeki (2004) also stated that, in the context of neuroaesthetics, ambiguity differs from uncertainty in that multiple valid solutions (interpretations) exist, but which is most valid remains undetermined. This definition is applicable not only to pictorial stimuli, but also to linguistic stimuli such as poetry in the present study. Such ambiguity, which generates and sustains multiple interpretations, is valued in art and literature and can lead to interest, engagement, and high evaluations (Armstrong, 2013; Muth et al., 2015). How, then, has the neural representation of such (poetic) textual ambiguity been examined?

Specifically, in empirical studies using fMRI, the processing of textual ambiguity, particularly semantic ambiguity, was consistently associated with increased activity in the left inferior frontal gyrus (IFG). According to Rodd et al. (2005), when processing sentences with high ambiguity containing polysemous words, the brain shows significant activity in the left IFG and the left posterior inferior temporal cortex in selecting and integrating contextually appropriate meanings from multiple competing semantic representations. Another study (Zempleni et al., 2007) compared sentences using the same polysemous words when the sentence-final context supported either the dominant or subordinate meaning; the latter showed stronger activation of the semantic control network centered on the left IFG and temporal lobe, as it required switching and reintegrating meanings. However, in haiku, ambiguity is thought to arise not only from lexical ambiguity but also from the diversity of interpretation created by omission of information and *toriawase* (取り合わせ: the technique of juxtaposing two distinct images to create a poetic resonance or spark between them).

Different neural substrates have been identified for such ambiguity and novelty of poetic language. Bohrn et al. (2013) showed that in the appreciation of texts with poetic features, the DMN, including the posterior cingulate cortex (PCC) and precuneus, showed stronger activation as familiarity with the text increased, suggesting that poetic comprehension involves self-referential processing and scenic recollection. Additionally, Mashal et al. (2009) reported that during the comprehension of novel metaphors extracted from poetry, the PCC, precuneus, left angular gyrus, and temporal lobe show specific activation, revealing that the interpretation of literary expressions involves substantial conceptual integration and introspective processing beyond the selection of existing lexical meanings.

### 1.4 Aims

Based on the above, the present study conducted the first cognitive neuroscience experiment in which participants appreciated haiku and provided aesthetic evaluations while inside an MRI scanner. The specific aims and hypotheses are as follows. First, as behavioral data, we compared beauty ratings between high- and low-ambiguity haiku. Consistent with previous studies (Hitsuwari and Nomura, 2024a,b), we predicted that low-ambiguity haiku would receive higher beauty ratings than high-ambiguity haiku. Second, we compared brain activity during the appreciation of haiku pre-categorized as having high or low ambiguity. We predicted that, depending on the level of ambiguity, brain regions related to semantic processing, such as the left IFG and left temporal lobe (Rodd et al., 2005; Zempleni et al., 2007), and the DMN, including the precuneus involved in self-referential processing and introspection (Bohrn et al., 2013; Mashal et al., 2009), would be activated. Third, we examined the relationship between the aesthetic evaluation of haiku and brain activity. We predicted that the more beautiful haiku are perceived to be, the greater the activation in brain regions associated with aesthetics, including the orbitofrontal cortex, bilateral insular cortex, left fusiform gyrus, reward networks including the caudate nucleus and nucleus accumbens (Bohrn et al., 2013; Gao and Guo, 2018; Wassiliwizky et al., 2017), and the occipital pole and DMN related to visual processing and mentalizing (Kumar et al., 2025; Zeman et al., 2013). Fourth, using brain regions activated during low- and high-ambiguity haiku as seeds, we examined functional connectivity using aesthetic evaluations. Finally, we examined whether brain regions showing differential activity between high- and low-ambiguity conditions retained neural patterns related to ambiguity. We used multivoxel pattern analysis (MVPA) to assess whether these regions could discriminate between low- and high-ambiguity haiku.

## 2 Materials and methods

This study was approved by the Ethics Committee of the Graduate School of Education, Kyoto University (CPE-510), and the Safety Review Committee of the Institute for the Future of Human Society MRI Research Facility, Kyoto University (22-003). All procedures were carried out in accordance with the relevant guidelines and regulations, including the Declaration of Helsinki.

### 2.1 Participants

Forty-four right-handed participants without neurological or psychiatric disorders were recruited via the electronic bulletin board at Kyoto University. After excluding five participants because of data-quality issues, data from 39 participants (M_age_ = 21.69, SD = 1.64, 24 men and 15 women) were included in the analyses. Normal or corrected-to-normal vision was confirmed before the experiment. The target sample size was determined *a priori* using a power analysis (pwr package in R; Champely, 2020; R Core Team, 2025), assuming a medium correlation (*r* = 0.40), *α* = 0.05, and power = 0.80, which yielded *n* ≈ 46. The final analyzed sample (*N* = 39) was comparable to or larger than those used in previous fMRI studies of poetry appreciation (Wassiliwizky et al., 2017; Zeman et al., 2013).

### 2.2 Stimuli

Haiku were selected from an anthology (Kadokawa Gakugei Publishing, 2008) containing highly acclaimed haiku from Japan. A preliminary survey was conducted with 169 participants (Mage = 39.27, SD = 10.44; 78 women, 91 men) who did not participate in the main experiment. Participants were limited to those with university or graduate school degrees or current university or graduate students, and were recruited through CrowdWorks,^1^ a Japanese online survey platform. Of the 680 haiku, each participant evaluated 40 poems. Participants responded to the question “How much ‘ambiguity’ did you feel when reading the following haiku?” on a scale of 1 (“not at all”) to 7 (“very much”). We selected 48 haiku rated as higher in ambiguity (M_ambiguity_ = 5.32, SD = 0.19, Range = 5.09–5.75) and 48 haiku rated as lower in ambiguity (M_ambiguity_ = 2.31, SD = 0.30, Range = 1.56–2.73). These high- and low-ambiguity haiku showed significant differences in ambiguity scores [Welch’s *t*(80.12) = 59.37, *p* < 0.001, Cohen’s *d* = 12.12, 95% CI (10.31, 13.86)]. Representative examples of the haiku used in each condition are shown in Table 1. In the present study, we created two lists using half of these high- and low-ambiguity haiku, and the participants appreciated and evaluated one list.

**Table 1:** Representative haiku stimuli for the high- and low-ambiguity conditions.

Condition	Haiku (original)	Romanization	English (Authors’ translation)	Mean ambiguity
High ambiguity	湯豆腐やいのちのはてのうすあかり	yudōfu ya inochi no hate no usuakari	Simmered tofu: a faint light at the very end of life	5.73
Low ambiguity	六月を綺麗な風の吹くことよ	rokugatsu o kirei na kaze no fuku koto yo	In June, how lovely the wind that blows	2.70

One example per condition. Mean ambiguity ratings are from an independent pilot norming study. Both poems are in the public domain (high ambiguity: by Kubota Mantarō, d. 1963; low ambiguity: by Masaoka Shiki, d. 1902). Translations by the authors.

### 2.3 Procedure

Upon arriving at the laboratory, the participants received experimental instructions, including MRI-related precautions, and signed an informed consent form. After viewing the task flow to appreciate and evaluate the haiku and completing several practice trials, participants entered the MRI scanner. Each trial consisted of a 1-s fixation point, a 1-s instruction (“Please evaluate the ‘beauty’ of the following haiku”), a 0.5-s fixation point, 6 s of haiku presentation, 3.5 s for evaluation, and a 3-s interstimulus interval (during which “The next evaluation will begin in 3 s” was displayed), with 48 consecutive trials. The haiku were evaluated on a 4-point scale from 1 (“I feel no beauty at all”) to 4 (“I feel a great sense of beauty”).

Several tasks were also included for the purposes of another study. First, a task evaluating three types of ambiguity (imagery, interpretation, and emotional ambiguity) was conducted beforehand. In this task, haiku were presented progressively by dividing them into lines 1, 2, and 3 to track changes in ambiguity [a similar procedure was used in Hitsuwari and Nomura (2025b)]. In addition, participants completed several individual trait questionnaires. These were distributed via email to be completed on the day of the experiment, thereby reducing the response burden. However, these results are not reported in the present study. A 6-min eyes-open resting-state scan was also acquired in the same session and, together with one of these questionnaires (a measure of attitudes toward ambiguity), was analyzed in a separate preregistered report (Hitsuwari, 2026).

### 2.4 MRI setting

MRI images were acquired using a 3-Tesla MRI scanner (Siemens Verio, Germany) owned by the Institute for the Future of Human Society, Kyoto University, with a 32-channel head coil. The brain images were acquired using multiband imaging. Acquisition parameters were as follows: multiband factor 4, 76 slices, slice thickness 2.0 mm, slice gap 0.0 mm, voxel size 2 × 2 × 2 mm, flip angle 80°, repetition time (TR) 2000 ms, echo time (TE) 43 ms, field of view (FoV) 192 mm, and acquisition matrix 96 × 96. T1-weighted images were acquired using a magnetization-prepared rapid acquisition gradient echo (MPRAGE) sequence. Acquisition parameters for whole-brain coverage were: 208 slices, slice thickness 1.0 mm, slice gap 0.0 mm, voxel size 1.0 × 1.0 × 1.0 mm, flip angle 9°, TR 2250 ms, TE 3.51 ms, FoV 256 mm, and acquisition matrix 256 × 256. Haiku stimuli were presented using a mirror attached to the head coil. The first four images were excluded from subsequent analyses because of noise and data instability.

### 2.5 fMRI data analysis

#### 2.5.1 Pre-processing of fMRI data

Pre-processing of functional and structural brain images was performed using Statistical Parametric Mapping 25 (Wellcome Centre for Human Neuroimaging, 2025) in MATLAB R2024b (The MathWorks Inc., 2024). First, a slice-timing correction was applied to the acquired functional images using the 38th slice as the reference, followed by motion correction. The correction employed a rigid-body transformation with translation in the superior–inferior, left–right, and anterior–posterior directions, as well as rotation around the superior–inferior, left–right, and anterior–posterior axes. Each participant’s structural image was normalized to the Montreal Neurological Institute (MNI) space, and gray matter, white matter, and cerebrospinal fluid images were segmented and extracted. To align the motion-corrected functional images with the segmented structural images, a rigid-body transformation was used to coregister the mean functional image of each participant with their structural image. The coregistered functional images were anatomically normalized using a template. Spatial smoothing was performed using an 8 × 8 × 8 mm Gaussian filter. Spatial normalization was also applied to the segmented structural images. A high-pass filter (cut-off: 128 s) was applied to the time series of each voxel to remove low-frequency noise.

#### 2.5.2 First-level analysis

For each participant, brain activity was estimated using a general linear model. For the ambiguity contrast analysis (low ambiguity vs. high ambiguity), the onset of each trial was defined as an event using a 6-s boxcar function. For the parametric modulation analysis of the beauty ratings, the beauty rating value for each trial (within-subject centered) was used as a regressor and convolved with the standard hemodynamic response function (HRF). In addition, six motion parameters were included as covariates.

#### 2.5.3 Second-level analysis

One-sample *t*-tests were used for group-level statistical analyses (*n* = 39). For whole-brain analysis, the voxel-level threshold was set at *p* < 0.001 (uncorrected), and the cluster-level threshold was set at *p* < 0.05 (family-wise error rate–corrected: FWE-corrected). To assess whether the low > high ambiguity contrast was confounded by aesthetic appreciation, we estimated an additional whole-brain GLM in which trial-wise beauty ratings were entered as within-condition parametric modulators (mean-centered and without orthogonalization). Because the modulators were mean-centered within each condition, the low > high ambiguity contrast was orthogonal to the beauty regressors, allowing the ambiguity effect to be evaluated after accounting for beauty ratings.

To confirm that the cluster-level findings were not dependent on Gaussian random field assumptions, we additionally performed nonparametric permutation testing using SnPM13 (Statistical nonParametric Mapping; Nichols and Holmes, 2002). For each whole-brain analysis, the sign of each participant’s contrast image was randomly flipped across 10,000 permutations (without variance smoothing), and family-wise error was controlled using the permutation distribution of the maximum statistic. We report peak-level (voxel-wise) and cluster-level FWE-corrected results (cluster-forming threshold *p* < 0.001), together with Cohen’s *d* effect-size maps.

#### 2.5.4 Psychophysiological interaction (PPI) analysis

To examine whether beauty ratings modulated functional connectivity, we conducted an exploratory PPI analysis. As seed regions, we used the PCC [2, −28, −8] and the left inferior parietal lobule (IPL) [−60, −42, 40] obtained from the ambiguity contrast, and the right V1/V2 [14, −82, 2] obtained from the parametric modulation of beauty. A 6-mm spherical volume of interest (VOI) was defined around each seed, and the BOLD time series was extracted. BOLD signals were converted to neural activity signals by HRF deconvolution using Tikhonov regularization (*λ* = 1.0). Beauty rating values (within-subject centered) were placed on the TR grid as a 6-s boxcar function to serve as the psychological variable. The PPI regressor was created by convolving the element-wise product of the neural activity signal and psychological variables with HRF. Each participant’s first-level GLM included physiological variables (seed activity), psychological variables (beauty ratings), PPI variables (interaction terms), and six motion parameters as regressors (high-pass filter, 128 s; no orthogonalization). At the group level, one-sample *t*-tests were conducted for PPI > 0 contrast, with cluster-level FWE correction (*p* < 0.05, initial threshold *p* < 0.001 uncorrected).

#### 2.5.5 Multivoxel pattern analysis (MVPA)

To explore whether the brain regions associated with ambiguity encode generalizable neural patterns, we performed MVPA. BOLD signals were extracted from 6-mm spherical regions of interest (ROIs) centered on the PCC [2, −28, −8] and left IPL [−60, −42, 40], using mean values from 4 to 10 s post-stimulus onset, and reduced to ten dimensions via principal component analysis. The ambiguity conditions (low versus high) were classified using leave-one-trial-out cross-validation and linear support vector machines (SVMs).

## 3 Results

### 3.1 Behavioral results

**Figure 1:**
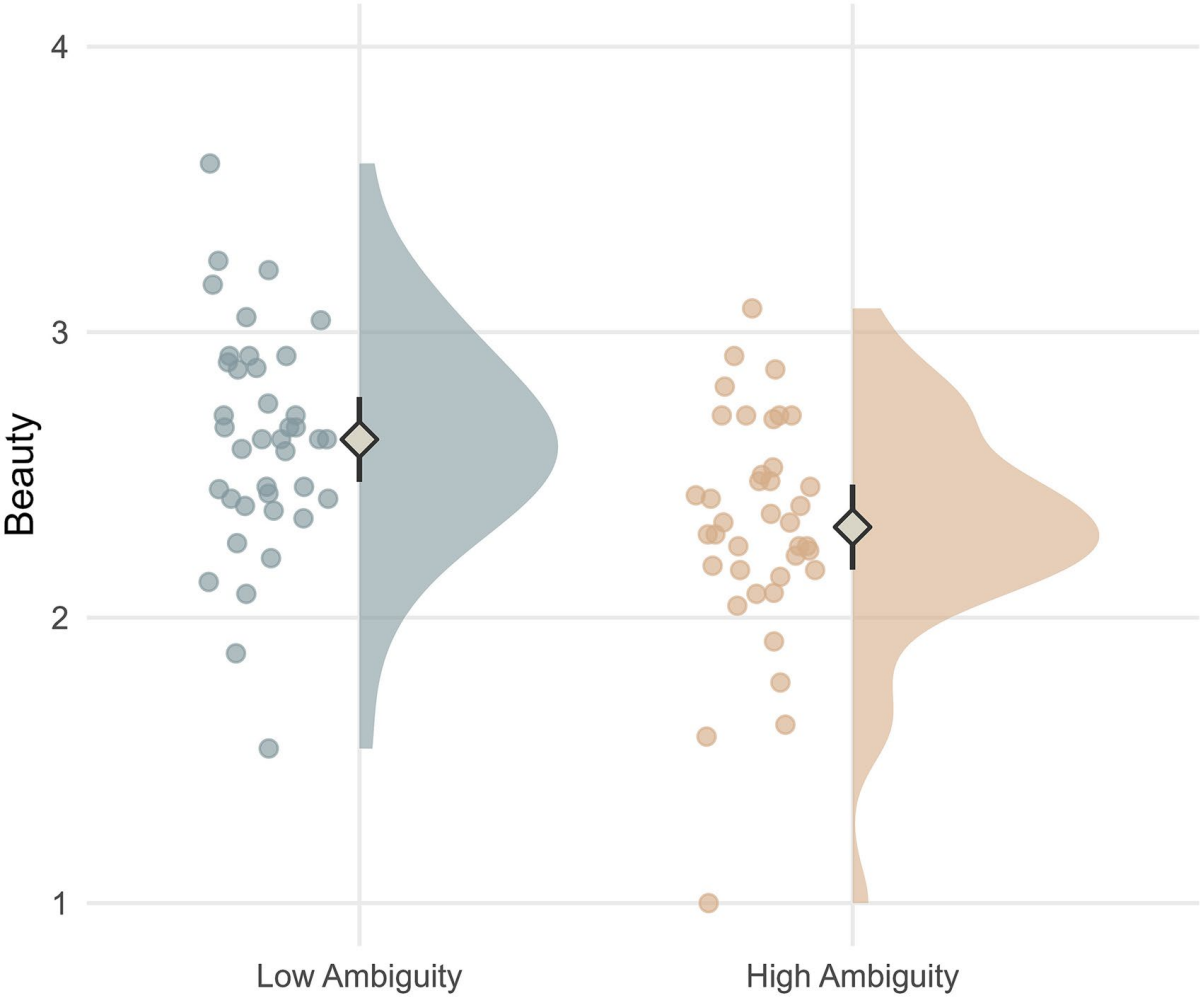
Beauty ratings by ambiguity condition. For each condition, the density of participants’ mean ratings (*n* = 39; each point = one participant) is shown with the model-estimated marginal mean and 95% CI from a linear mixed-effects model (condition as a fixed effect; participants and haiku as crossed random effects).

The effect of the ambiguity condition on the aesthetic evaluation of haiku was examined using a linear mixed-effects model, with participants and haiku as random effects. The main effect of ambiguity condition was significant in the model [*β* = −0.31, *SE* = 0.09, *t*(94.01) = −3.56, *p* < 0.001, standardized *β* = −0.16, 95% CI (−0.25, −0.07)], with high-ambiguity haiku receiving significantly lower aesthetic evaluations than low-ambiguity haiku. Descriptive statistics showed means for low ambiguity (M = 2.62, SD = 0.97) and high ambiguity (M = 2.32, SD = 0.90) (Figure 1) with a small effect size. The model fit statistics were conditional *R*^2^ = 0.268 and marginal *R*^2^ = 0.026, indicating that the overall model combining fixed and random effects explained 26.8% of the variance, whereas the fixed effect of the ambiguity condition alone explained 2.6%.

### 3.2 Brain results

**Figure 2:**
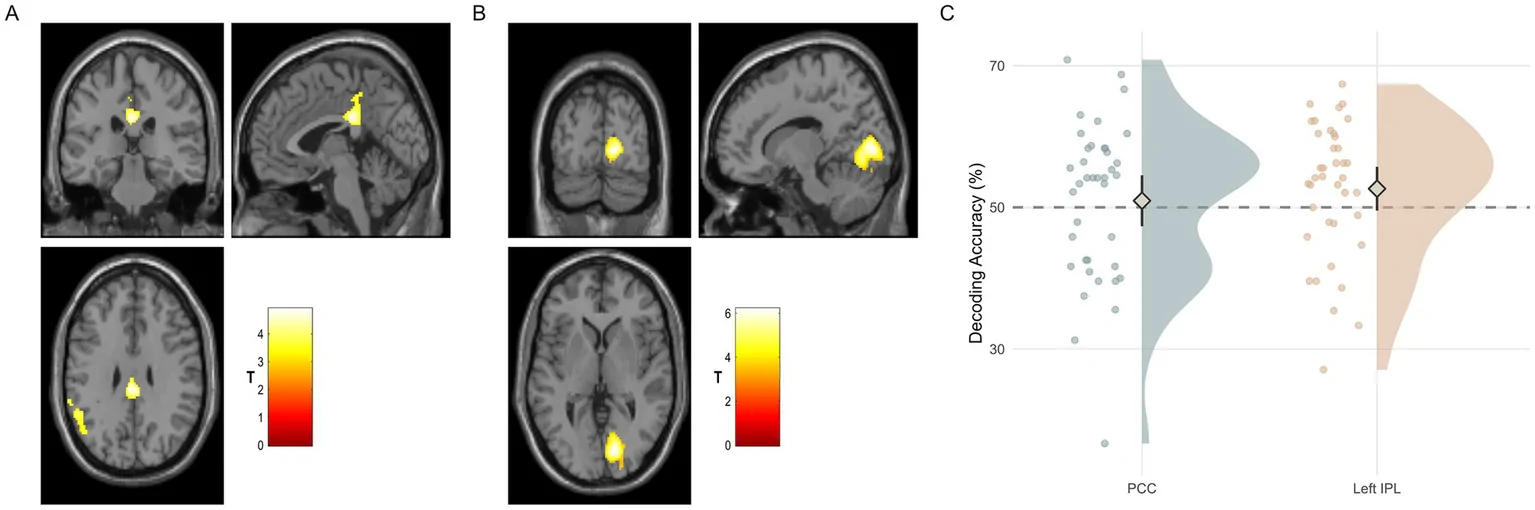
Neural correlates of ambiguity and beauty in haiku processing. **(A)** Whole-brain analysis revealed greater activation for low- vs. high-ambiguity haiku in the PCC/precuneus and left IPL. **(B)** Beauty ratings positively modulated activity in right calcarine cortex (V1/V2). **(C)** MVPA decoding accuracy in the PCC and left IPL. Each point = one participant (*n* = 39); diamond and error bar = group mean and 95% CI; dashed line = chance (50%).

First, a whole-brain analysis (*n* = 39) was conducted to examine the neural differences between low- and high-ambiguity haiku. Using a voxel-wise height threshold of *p* < 0.001 (uncorrected) and a cluster-level threshold of *p* < 0.05 (FWE-corrected), two significant clusters emerged for the contrast low > high ambiguity (Figure 2A). One significant cluster was located in the PCC, extending into the precuneus [peak MNI (−2, −28, 30), *T*(38) = 4.82, cluster *p*_FWE_ = 0.033, *k* = 411], and a second cluster was found in the left IPL, encompassing the supramarginal and angular gyri [peak (−50, −62, 24), *T*(38) = 4.54, cluster *p*_FWE_ = 0.005, *k* = 674; see Table 2]. Both survived the cluster-level corrections, indicating spatially extended effects. These findings were confirmed by nonparametric permutation testing: both clusters survived cluster-level FWE correction (PCC: cluster *p*FWE = 0.026; left IPL: cluster *p*FWE = 0.016), with permutation-based *p*-values equal to or smaller than the corresponding parametric estimates. At the peak (voxel) level, the PCC remained FWE-significant (*p*FWE = 0.035), whereas the left IPL peak was marginal (*p*FWE = 0.063). Peak effect sizes were large (Cohen’s *d* = 0.77 for both clusters). No significant clusters were found for the reverse contrast (high > low ambiguity) at the same threshold, even with a liberal voxel-wise criterion of *p* < 0.001 uncorrected. These findings suggest that reading a low-ambiguity haiku, which is semantically more interpretable, engages regions associated with semantic integration, self-referential processing, and conceptual imagery, including the PCC/precuneus and left IPL. After including trial-wise beauty ratings as within-condition parametric modulators, the left IPL cluster remained significant [cluster pFWE = 0.012, *k* = 560; peak at (−50, −62, 24), *T* = 4.46], whereas the PCC/precuneus cluster no longer survived cluster-level FWE correction [cluster pFWE = 0.075, *k* = 315; peak voxel unchanged at (−2, −28, 30), *T* = 4.82].

**Table 2:** Significant clusters from whole-brain analyses.

Contrast	Region	Hemisphere	*k*	*x*	*y*	*z*	*T*	*p*_FWE (param)	*p*_FWE (SnPM)	Cohen’s *d*
Low > high ambiguity	PCC/precuneus	L/midline	411	−2	−28	30	4.82	0.033	0.026	0.77
	Left IPL (SMG/angular)	L	674	−50	−62	24	4.54	0.005	0.016	0.77
Beauty (positive)	R calcarine (lingual/cuneus)	R	861	14	−82	2	6.15	0.002	0.011	0.98
	Secondary local maximum	R	—	12	−70	−6	5.58	—	—	—

Whole-brain one-sample t-tests (N = 39). Voxel-forming threshold p < 0.001 (uncorrected); cluster threshold p < 0.05 (FWE-corrected), whole brain. Coordinates are GLM cluster peaks in MNI space. p_FWE (param) = cluster-level FWE-corrected p from random field theory; p_FWE (SnPM) = cluster-level FWE-corrected p from 10,000 permutations (SnPM13). Cohen’s d is reported at the cluster peak. PCC = posterior cingulate cortex; IPL = inferior parietal lobule; SMG = supramarginal gyrus; L = left; R = right.

Second, a whole-brain one-sample *t*-test was conducted to identify brain activity that covaried positively with participants’ beauty ratings. One significant cluster emerged for the contrast beauty rating (positive modulation). This cluster was located in the right calcarine cortex, extending into the lingual gyrus and cuneus [peak MNI (14, −82, 2), *T*(38) = 6.15, cluster *p_FWE_* = 0.002, *k* = 861; Figure 2B; Table 2]. A secondary local maximum was found at [12, −70, −6], *T*(38) = 5.58. This cluster was likewise confirmed by nonparametric permutation testing (cluster *p_FWE_* = 0.011; peak *p_FWE_* < 0.001; Cohen’s *d* = 0.98). These findings suggest that higher subjective beauty ratings were associated with increased activation in early visual areas (V1/V2), indicating that even for language-based art forms such as haiku, enhanced visual imagery and perceptual encoding contribute to aesthetic experience.

Third, to explore whether beauty ratings modulated functional connectivity, we conducted PPI analyses using three seed regions: the PCC, left IPL (from ambiguity contrast), and right V1/V2 (from beauty parametric modulation). Only the V1/V2 seeds showed significant connectivity modulation. Specifically, set-level statistics revealed a significant negative modulation (*p* = 0.001), indicating that higher beauty ratings were associated with reduced connectivity between V1/V2 and distributed brain regions, particularly in the parietal and temporal areas. Although no individual clusters survived the cluster-FWE correction, the significant set-level statistics suggest a spatially distributed decoupling of V1/V2 from higher-order processing regions during the aesthetic experience. This effect reached significance only at the set level, indicating a distributed effect that cannot be localized to specific regions; it should therefore be regarded as preliminary. The PCC and left IPL, by contrast, showed no connectivity modulation based on beauty ratings (all *p* > 0.50).

Finally, we explored whether brain regions showing ambiguity-related activation encoded generalizable neural patterns using MVPA. BOLD signals were extracted from 6-mm spherical ROIs centered at PCC [2, −28, −8] and left IPL [−60, −42, 40], averaged across 4–10 s post-onset, and reduced to ten principal components. Leave-one-trial-out cross-validation with a linear SVM was used to classify the ambiguity conditions (low vs. high). For the left IPL, decoding accuracy was numerically above chance (M = 52.6%, SD = 9.5%, Cohen’s *d* = 0.28; one-tailed *t*(38) = 1.73, *p* = 0.042), whereas decoding in the PCC remained at chance (M = 50.9%, *t*(38) = 0.53, *p* = 0.30; Figure 2C). We note that the reported *p*-value is based on a one-tailed test against chance and that the left IPL effect does not survive either a two-tailed test or correction for the two ROIs examined (Bonferroni-corrected *α* = 0.025). We therefore interpret the MVPA findings as exploratory. The small left IPL effect is considered suggestive rather than confirmatory, whereas decoding in the PCC did not exceed chance.

## 4 Discussion

The present study is the first to examine brain activity and aesthetic evaluation during the appreciation of haiku using an MRI scanner. At the behavioral level, consistent with previous studies (Hitsuwari and Nomura, 2024a,b), low-ambiguity haiku were evaluated as slightly more beautiful than high-ambiguity haiku. Although the effect size was small, the tendency for haiku with relatively clear meanings and imagery to receive higher beauty ratings (Belfi et al., 2018; Hitsuwari and Nomura, 2022; Mehl et al., 2025) was replicated even in the MRI environment. The ambiguity condition nonetheless explained only 2.6% of the variance in beauty ratings (marginal *R*^2^ = 0.026); this preference should therefore be regarded as a subtle, group-level tendency rather than a strong determinant of aesthetic evaluation.

### 4.1 Interpretive ambiguity and self-referential integration

Whole-brain analysis of the ambiguity contrast revealed significantly higher activity in the PCC/precuneus and the left inferior parietal lobule (including the supramarginal gyrus and angular gyrus) in the low-ambiguity > high-ambiguity condition. The PCC/precuneus, as a core component of the DMN, is involved in autobiographical memory, self-referential processing, and internal scene simulation (Bohrn et al., 2013; Zeman et al., 2013), whereas the left IPL is considered a higher-order semantic processing region responsible for lexical and conceptual integration and metaphorical meaning comprehension (Mashal et al., 2009).

Lexical ambiguity, typically examined in general language research, occurs when a word has multiple meanings, with sense selection and inhibition centered on the semantic control network involving the left IFG (Rodd et al., 2005; Zempleni et al., 2007). In contrast, the haiku in this study involve interpretive ambiguity, where multiple scenes and interpretations may arise through juxtaposition and omission. Readers are not choosing among word senses but constructing a particular scene or narrative (see Table 1). Contrary to our prediction, we did not observe the hypothesized involvement of the left IFG; no ambiguity-related difference in this region reached significance, even at the voxel-forming threshold. The finding that ambiguity-related differences emerged in the PCC/precuneus and left IPL rather than the left IFG suggests that ambiguity in haiku is resolved through self-referential and integrative processes involved in constructing a situational representation, rather than through word-sense selection. Low-ambiguity haiku may be associated with readers’ construction of a single coherent scene or narrative, accompanied by stronger activation in the PCC and left IPL. Because participants had already read each haiku in the preceding ambiguity-evaluation task, however, the PCC/precuneus findings should be interpreted with caution. These regions are strongly implicated in familiarity and autobiographical memory retrieval; consequently, part of the observed effect may reflect greater familiarity with the more memorable low-ambiguity haiku rather than ambiguity processing alone. Although the left IPL ambiguity effect remained significant after controlling for beauty, the PCC/precuneus effect no longer survived cluster-level correction. This finding suggests that PCC activity in the low > high ambiguity contrast may reflect variance shared with aesthetic appreciation, familiarity, or memory-related processing rather than ambiguity alone. More broadly, because both the PCC/precuneus and the left IPL are functionally heterogeneous regions engaged by semantic integration, episodic and autobiographical memory retrieval, and attentional control, the present effects cannot be attributed uniquely to ambiguity, even where they remained statistically robust after controlling for beauty.

No significant activity differences were detected in the reverse contrast (high ambiguity > low ambiguity). In high-ambiguity haiku, participants may abandon active meaning construction because of sparse semantic cues and instead shift to a more casual reading strategy. Therefore, neither the frontal lobe load for controlling semantic competition nor self-referential and scenic processes may have been sufficiently activated.

The exploratory MVPA findings are consistent with this interpretation. Decoding accuracy in the left IPL was numerically above chance, although the effect size was small, whereas decoding in the PCC remained at chance. Because the left IPL effect did not survive correction for the two ROIs, we interpret it as suggestive rather than confirmatory. Nevertheless, it is directionally consistent with the robust univariate findings in the left IPL. The left IPL may be more sensitive to how readily a haiku coheres with meaning and scene, whereas the PCC, which is more broadly involved in self-referential introspection and memory retrieval, may not necessarily distinguish fine-grained ambiguity differences.

In literary and artistic theory, ambiguity has been valued as a form of play that allows movement between multiple interpretations (Armstrong, 2013; Empson, 1949; Muth et al., 2015). The results of the present study suggest that not all ambiguities are welcomed: activation in the DMN and left IPL, along with higher aesthetic ratings, tended to be greater when ambiguity remains foreseeably interpretable while allowing room for interpretation. This pattern is broadly consistent with proposals that the DMN contributes to aesthetic appeal as part of a sense-making system that is engaged when ambiguity can be resolved through integration rather than by ambiguity per se (Vessel and Ovadia, 2025; Vessel et al., 2019). We therefore interpret the observed DMN involvement cautiously, suggesting that a moderate, resolvable degree of ambiguity may preferentially engage the integrative and self-referential processes associated with these regions.

### 4.2 Beauty and visual mental imagery

Parametric modulation analysis of beauty ratings revealed that activity in the right primary and secondary visual cortices (calcarine cortex, lingual gyrus, and cuneus) was positively associated with aesthetic evaluation. This indicates that even in haiku, a linguistic art form, how clearly a scene is “seen” is strongly linked to subjective beauty.

Studies examining Chinese (Gao and Guo, 2018) and Sanskrit poetry (Kumar et al., 2025) also reported the recruitment of occipital visual areas during poetic comprehension and aesthetic experiences. Additionally, the fact that the imagery vividness of haiku and poetry predicts aesthetic preference has been consistently shown at behavioral and physiological levels (Belfi et al., 2018; Chaudhuri and Bhattacharya, 2025a; Hitsuwari and Nomura, 2022; Mehl et al., 2025). The results of the present study support these findings, suggesting that even with extremely brief texts, such as haiku, activity in the primary visual cortex is associated with aesthetic appreciation and may reflect the engagement of visual imagery during reading. More broadly, recent reader-response research has begun to distinguish aesthetic and immersive processing during literary reading. Using eye-tracking, Tilmatine et al. (2026) showed that these processes are dynamically intertwined rather than mutually exclusive and argued that behavioral measures alone cannot determine their neural basis, highlighting the need for neuroimaging approaches. The present fMRI findings contribute to addressing this gap by situating the visual and self-referential correlates of haiku appreciation within this broader framework of literary reading. Taken together, our findings suggest that the aesthetic experience of haiku is associated with both semantic integration involving the DMN and visual activity in the occipital cortex.

### 4.3 Functional connectivity and the absence of reward-system involvement

The PPI results also align with this interpretation. Set-level statistics suggested that functional connectivity between the V1/V2 and parietal–temporal regions weakened as beauty ratings increased. No individual clusters survived cluster-level FWE correction, so these results are statistically weak and exploratory, and the following interpretation is offered tentatively. The reduced connectivity may be consistent with a shift of the visual cortex toward internally generated imagery rather than the processing of external input, although this possibility requires confirmation in future, hypothesis-driven studies. During the reading of poetry and narratives, regions belonging to the default mode network, such as the cingulate cortex and hippocampus, show strong activation, evoking autobiographical memory and imaginative thinking (Zeman et al., 2013). The present study extends this internal simulation process to haiku.

However, we did not detect an association between aesthetic evaluation and reward system activity (mOFC, nucleus accumbens, and others). This may reflect the stimulus set: all haiku were moderately pleasing, making them unlikely to evoke the intense pleasure or chills often reported in visual art or listening to extended poetry (Ishizu and Zeki, 2011; Wassiliwizky et al., 2017).

This study is notable for examining the neural basis of ambiguity and aesthetic experience using haiku. In particular, it was shown that more interpretable haiku elicited stronger activation in the PCC/precuneus and left IPL and that subjective beauty was associated with increased activity in the primary and secondary visual cortices. These results suggest that aesthetic experience in haiku appreciation is supported by interpretable ambiguity and visual mental imagery. Haiku also continues to spread across cultures and media, including digital platforms (Meilantari et al., 2024), and the present study contributes a neuroscientific perspective to this growing body of research.

### 4.4 Limitations

This study had several limitations. First, the participants in the present study were limited to Japanese university students and the results cannot be generalized to haiku experts or readers from other cultures. Previous research has shown that culture and expertise modulate the relationship between ambiguity and aesthetic evaluation (Hitsuwari and Nomura, 2024a,b). In the future, it is expected that similar paradigms will be applied to expert readers and cross-cultural samples to examine whether interpretive ambiguity produces comparable behavioral effects and patterns of activity within the DMN and semantic networks.

Second, because the behavioral effect of ambiguity on beauty ratings was small (marginal *R*^2^ = 0.026), the observed preference should be treated as a subtle, group-level tendency rather than a strong or general determinant of individual aesthetic judgments.

Third, both the PPI and MVPA analyses conducted in the present study used post hoc seeds and ROIs based on contrast results derived from this study and were not confirmatory analyses using independently established regions of interest. Therefore, while decoding ambiguity using the left IPL and connectivity changes in V1/V2 accompanying aesthetic evaluation are suggestive, they cannot be considered definitive evidence of which regions code for ambiguity or aesthetic experience. More generally, because the PCC/precuneus and left IPL are engaged by many cognitive processes beyond ambiguity, and because the PCC/precuneus effect did not survive control for beauty ratings, the whole-brain activations reported here should likewise not be read as evidence that these regions specifically or uniquely encode ambiguity.

Finally, in the present study, haiku were presented line by line in the ambiguity-evaluation task prior to the aesthetic evaluation task; therefore, the haiku during aesthetic evaluation was not completely novel. Familiarity with poetry has been associated with DMN activity and preference (Bohrn et al., 2013). Therefore, the activity in the PCC/precuneus and left IPL and the aesthetic ratings observed in the present study may include familiarity from repeated reading.

## 5 Conclusion

Overall, the present study provides initial evidence of how interpretive ambiguity and beauty are represented in the brain when reading haiku, an extremely condensed form of literature. The central finding is that the ambiguity of haiku was associated with the PCC/precuneus and left IPL rather than the left IFG, suggesting that, unlike the word-sense ambiguity examined in psycholinguistics, interpretive ambiguity is handled through self-referential and integrative processing that supports the construction of a situational world. Subjective beauty, in turn, was accompanied by activity in early visual areas, consistent with the engagement of visual mental imagery during reading. Because the connectivity and decoding evidence was exploratory, these interpretations await confirmation in future hypothesis-driven and cross-cultural studies. More broadly, the fixed form of haiku makes it a compact and well-controlled model for isolating interpretive ambiguity and aesthetic response, and such a model offers a foundation for advancing neuroscientific investigations of ambiguity and aesthetic experience in poetry, narratives, and other art forms.

## Data Availability

Individual-level MRI data are not publicly available due to participant privacy considerations but may be shared upon reasonable request to the corresponding author. Analysis scripts (including the nonparametric SnPM scripts) are available on OSF at https://osf.io/emsp4/. The permutation analyses used SnPM13.1.09 under SPM25 and MATLAB R2024b.

## Footnotes

1
https://crowdworks.jp/
